# Equity Structure, Strategic Investment Psychology, and Performance in China’s Green Economy Context

**DOI:** 10.3389/fpsyg.2021.707582

**Published:** 2021-08-25

**Authors:** Li Xin Guo, Kuen-Lin Lin, Li-Ting Zhang, Chi-Fang Liu

**Affiliations:** ^1^School of Business, Huaiyin Institute of Technology, Huai’an, China; ^2^Department of Business Administration, Cheng Shiu University, Kaohsiung, Taiwan

**Keywords:** equity structure, strategic investment psychology, green economy, largest shareholder, green affair investments

## Abstract

This study empirically tests the impacts of equity structure on strategic investment psychology in green affairs in R&D vs. Marketing dimensions and company performance. Based on data from Chinese high-tech industry listed companies, the empirical results show that: (1) the largest shareholder’s shareholding ratio has a positive effect on marketing investment psychology and a negative impact on R&D investment psychology, (2) other large shareholders’ shareholding ratio are positive related to R&D investment psychology; (3) R&D investment psychology has a negative effect and marketing investment psychology has a positive influence on the current performance; (4) equity counterbalance is positive related to R&D investment psychology and has a negative effect on the current performance. This study contributes to the literature of corporate governance on sustainability issue by providing a new psychological perspective. The results also provide an important guidance for the corporate governance practice in green economies.

## Introduction

Existing literatures have done a lot of researches on the impact of equity structure on strategic investment and company performance. However, these literatures rarely specifically study strategic investments from psychological perspective in green affairs. For example, many scholars have studied the relationship between equity structure and corporate financial performance (Demsetz and Villalonga, 2001; Abdallah and Ismail, 2017; Ducassy and Guyot, 2017; Paniagua et al., 2018), and other scholars have studied the impact of equity structure on R&D investment. For example, previous studies argue that ownership structures have different characteristics and influences on R&D investment in different cultures and institutional contexts (Lee and O’neill, 2003; Lee, 2005), such as Lee and O’neill (2003) find that ownership concentration have a positive impact on R&D investment in the United States and no significant influence in Japan. Based on the sample of the listed companies in 19 European countries, Lopez Iturriaga and López-Millán (2017) find that ownership concentration have a positive effect on R&D investment in the countries with poor legal protection of investors.

Similarly, Baysinger et al. (1991) report that concentration of equity positively affect R&D spending, Francis and Smith (1995) find that concentrated ownership has promoted innovation, by using Spanish data, Tribo et al. (2007) showed that the number of block-holders had negative influence on R&D investment. Unlike these findings, Yafeh and Yosha (2002) report a negative relationship between ownership concentration and R&D expenditure, Holderness and Sheehan (1988) find that ownership concentration was not significantly correlated with R&D expenditure. Unfortunately, little literature pays attention to the impact of ownership structure on marketing investment, only a few studies have analyzed the influence of shareholder/investor types on marketing investment/orientation see Boo and Kim (2021), Qu et al. (2005), and Song et al. (2015).

Overall, existing research results show that the impact of equity structure on strategic investment and performance is related to institutional and cultural background. However, we do not know whether these research conclusions are applicable to strategic investment in green affairs. The changes in the global ecological environment continue to require manufacturing companies to take a green and sustainable development path. For example, to reduce or eliminate the emission of toxic gases such as carbon dioxide and carbon monoxide, and to prevent the direct discharge of industrial wastewater that does not meet the standards and contains heavy metals into the river, lakes or surrounding fields, to promote the concept of green consumption and environmental protection to consumers. In such background, manufacturing companies need to continuously carry out technological innovation, improve product design and manufacturing processes, and increase strategic investment in technologies such as energy saving and low waste generation. In addition, manufacturing companies also need to increase investment in green marketing to reduce environmental pollution or damage from product consumption.

In recent years, China has formulated a national strategy for high-quality and green development, and established new laws and regulations to guide manufacturing companies to choose sustainable green development strategies, encourage manufacturing companies to develop energy-saving and low-carbon emission prevention technologies, and support a circular economy for waste recycling and utilization. However, green investment based on ecological protection will increase the operating costs of enterprises, and there are certain risks. This makes many Chinese companies take a conservative attitude in increasing strategic investment in green affairs. We do not know what kind of equity structure will help manufacturing companies make strategic green investment decisions. On the other hand, Chinese cultural and institutional characteristics are obviously different from that of developed countries, under the context of collectivism culture and inadequate external institutions (Chen V.Z. et al., 2014; Zhang et al., 2014), Chinese companies generally have concentrated ownership, the largest shareholder owns a high proportion of shares beyond other large shareholders, and has strong control over the company. Moreover, the ties play an important role in Chinese companies, which can influence individual and group behaviors, the strong ties between the largest shareholder and top managers may eliminate the traditional agency problem, because the interests of the largest shareholders and top managers may be aligned. Therefore, in China, the principal-agent problem may mainly come from the inconsistency of interests between the largest shareholder and other large shareholders or small shareholders, and we believe that such characteristics of ownership structure have an important impact on Chinese companies’ strategic green investment decisions.

In the new era where China emphasizes green and high-quality development, this research integrates R&D investment and marketing investment into a unified theoretical framework, and studies the impact of equity structure on strategic investment from a psychological perspective, which is a further extension, deepening and supplement to existing research. It not only changes the research perspective and background of the existing literature, but also can improve the theoretical explanation ability (Under the constraints of the company’s resources, there is a trade-off between R&D investment and marketing investment).

## Theoretical Background and Hypothesis

### Theories

Agency theory assumes that there is an interest conflict between managers and dispersive shareholders (Berle and Means, 1932), the principal-agent problem induced by the excessive dispersion of ownership may be harmful to firms (Jensen and Meckling, 1976), because the separation of ownership and control may make managers to pursue self-serving priorities (Tribo et al., 2007), and to extract private benefits from firm resources (Shleifer and Vishny, 1986). One prescription for the PA conflict is to increase the concentration of ownership, the existence of large shareholders is conducive to monitor managers’ opportunistic behaviors and reduce agency costs (Dyck and Zingales, 2004). However, with the increase of ownership concentration, conflicts may arise between controlling shareholders and other shareholders (La Porta et al., 1999; Barca and Becht, 2001), because large shareholders tend to expropriate the benefits of minority shareholders (Tribo et al., 2007).

As an alternative perspective, stewardship theory supposes that the interests of managers may be consistent with those of shareholders (Fox and Hamilton, 1994; Davis et al., 1997), when managers are self-actualizing and have high organizational identification (Mael and Ashforth, 1992), their behaviors (termed “steward behavior”) may show the features of pro-organization and collectivist (Lee and O’neill, 2003). In collectivist and high power distance cultures, the steward behavior has a high incidence (Davis et al., 1997; Lee and O’neill, 2003).

China is a country where collectivism and high power distance culture prevail, at this point, it is similar to Japan. However, although the level of trust within a interest-groups is high, the level of trust between different groups is low. For Chinese listed companies, the largest shareholder and other large shareholders often have different interests and pursuits, thus, the agency problem are mainly reflected in the conflicts between other large shareholders and the largest shareholder. For the purpose of control and supervision, the largest shareholder usually appoints his relatives and friends as senior executives of listed companies, thus, the relationship between the largest shareholder and top managers is manifested in a steward form, and the relationship between other large shareholder and top managers has a principal-agent characteristics. For this reason, this study integrates agency theory and stewardship theory to analyze the influence of the largest shareholder and other large shareholders on strategic investment psychology in green affairs and performance.

### The Largest Shareholder

In China, the equities of the listed companies are relatively concentrated, and the largest shareholder has strong control over the company. For example, the largest shareholders can appoint their trusted relatives or friends as executives, so the relationship between the largest shareholder and top managers can be regarded as a “steward relationship,” the responsibility of top managers is mainly to fulfill the strategic intent of the largest shareholder. The more shares the largest shareholder has, the greater their control over the company, the more it can weaken the adverse effects of the principal-agent problems, and thus have a positive impact on the company’s performance.

In addition, during the reform of China’s economic system, changes in economic policies often offer many investment and market opportunities. Because such opportunities are time-sensitive, companies need fast and decisive strategic decisions/behaviors to seize this opportunity. The higher the control of the largest shareholder over the company, the better it is for the company to seize these opportunities. Therefore, the higher the shareholding of the largest shareholders, the stronger their controlling abilities, and the faster they can respond to new investment opportunities, and improve the company’s short-term performance.

Finally, we believe that the largest shareholders have a strong incentive to pursue good short-term performance, because the largest shareholders can gain extra benefits by manipulating short-term financial performance. In the Chinese stock market, the stock price is very sensitive to changes in the company’s financial performance, a slight increase in the company’s financial performance may lead to a substantial increase in the stock price, thus the largest shareholders can raise their stock price through improving short-term financial performance, and entrust other investors to buy and sell stocks for their private benefits. Therefore, we hypothesize:

*Hypothesis 1.1: In China, the shareholding ratio of the largest shareholder is positively related to the current performance.*

Under the environment of imperfect market economy system, the competitive advantage of listed companies mainly comes from the control of various market and government resources, it is important for listed companies to allocate more resources to various marketing activities. Especially by increasing investment in green marketing, the company’s social reputation can be directly and effectively improved, and a good impression can be established in the minds of customers and the government. Compared with R&D investment, the company’s investment in green marketing will improve its reputation more directly and quickly. This is because the payoffs from R&D in energy-saving, emission-reduction and environmental protection technologies generally take longer to gain. Relatively, the company’s contribution to social development, ecological-environmental protection, green product packaging, recycling, green consumption, etc., are all easier to be seen by stakeholders. Therefore, marketing investment in green affairs may create an immediate profit and improve company short-term performance, and the largest shareholder may like to invest in green marketing activities, in order to establish and maintain a good social image in the minds of the government and the public. Through the above analysis, we believe that the largest shareholder often handles the company’s strategic green marketing investment decision-making issues with a relatively optimistic and positive mentality. If the largest shareholder has a positive sentiment or psychological state for green marketing investment, it will affect the company’s strategic decision-making team members to have the same or similar psychology in green marketing investment. Obviously, the higher the shareholding ratio of the largest shareholder, the greater its influence on the company’s strategic decision-making psychology, so the shareholding ratio of the largest shareholder may have a positive impact on the company’s green marketing investment psychology. Therefore, we hypothesize:

*Hypothesis 1.2: In China, the shareholding ratio of the largest shareholder is positively related to the psychology of green marketing investment.*

As mentioned above, the largest shareholder has the psychological motivation to improve the company’s short-term performance, and may prefer to invest in various marketing activities. This investment psychology will affect and spread to the company’s strategic decision-making team, and promote the company’s strategic decision-makers to increase marketing investment. As we know, marketing investment can improve the company’s brand image, increase customer satisfaction and loyalty, expand the company’s market space, establish better company-customer relationships, and increase the company’s market share and product sales revenue, ultimately, it will improves the company’s short-term financial performance. Therefore, if strategic decision makers are more positive attitude toward green marketing investment, the companies may have better short-term performance. Thus, we hypothesize:

*Hypothesis 1.3: In China, the green marketing investment psychology is positively related to the current performance.*

As stated above, the largest shareholder may influence company performance by influencing strategic decision makers’ psychology and behaviors. As a strategic decisions, R&D investment in green technologies has high risks, because green R&D projects in high-tech industries are usually long-run and need a lot of money, the outcomes of R&D investments in green technologies are neither immediate nor certain (Lee and O’neill, 2003), in emerging economies, inadequate external institutions (such as weak knowledge property-rights protection, inefficient factor markets…) have impeded firms’ innovation behaviors (Chen V.Z. et al., 2014), and this external institutional features have created higher risk and more uncertain in green R&D investment. Therefore, under a highly uncertain environment for R&D investment returns, although the largest shareholders may hope that their shares have a long-term value, they will still not support green R&D decisions with high-risk and high-investment, because they bear a higher stake than other large shareholders, especially when facing R&D investment decisions that affect the survival of the company, the largest shareholder becomes more conservative than other major shareholders.

Due to small shareholders’ weak control over the company, the strategic decision conflicts of listed companies mainly occur between the largest shareholder and other large shareholders, as Lee and O’neill (2003) noted, large shareholders have incentives to reduce information asymmetry, when large shareholders get more detailed information of R&D projects, they can better understand the failure risk of R&D projects, for the sake of the “double-edged sword” feature of R&D investment in green technologies, compared with other large shareholders, the largest shareholder bears the greatest risk of R&D investment, and may not support investing in the high-risk R&D projects. The higher the shareholding ratio of the largest shareholder, the greater the R&D risk they bear, and the more likely they are to hold a negative attitude toward R&D investment. Therefore, we believe that the largest shareholder’s shareholding may negatively influence the company’s green R&D investment psychology. Thus, we hypothesize:

*Hypothesis 1.4: In China, the shareholding ratio of the largest shareholder is negatively related to the R&D investment psychology.*

Technological innovation-oriented companies have a more positive R&D investment psychology, and usually prefer to increase R&D investment to gain a competitive advantage. However, the existing findings of R&D investment effect conclusions are not consistent. Some studies have found positive effects, while others have found negative effects. For the high-tech industry, the return on R&D investment generally does not occur in the current period, and it usually lags by a few years. Especially, the R&D investment in environmental protection and green technology would increase the company’s operating costs and reduce corporate profits in the short term, with even negative effects on company’s short-term sales revenue. Therefore, we believe that the effect of green R&D investment psychology on the company’s current performance is negative and hypothesize:

*Hypothesis 1.5: In China, the green R&D investment psychology is negatively related to the current company performance.*

### Other Large Shareholders

In the Chinese context, the existence of other large shareholders is a constraint on the largest shareholder, because the largest shareholders have greater control over the company, they often choose trusted relatives and friends as top managers of listed companies, so the interests of the largest shareholders and most top managers of listed companies are consistent. In order to prevent the opportunistic behavior of the largest shareholder, other large shareholders have a strong incentive to supervise the decisions and behaviors of the top managers of listed companies. The higher their shareholding ratio, the stronger their monitoring motivation and ability. Supervisory behavior can prevent erroneous decisions and behaviors to a certain extent, for example, reducing unnecessary reception expenditures, preventing excessive management expenditures, private use of buses, and unreasonable sales expenses. Therefore, the impact of other large shareholders on company performance may also be positive.

Several studies suggest that other large shareholders play an important role on monitoring the selfish behavior of the largest shareholders and improving corporate governance, Pagano and Roell (1998) argue that other large shareholders can monitor and restrict the biggest shareholders’ opportunistic behaviors, Bloch and Ulrich (2001) show that the second large shareholders can reduce the diversion of company resources when their shareholding ratios are sufficiently large (Bloch and Ulrich, 2001; Laeven and Levine, 2004), Luc Laeven and Ross Levine (2005) further claim that other large owners can improve corporate governance and valuations (Laeven and Levine, 2004). Furthermore, from the perspective of monitoring incentive and collusion incentive, Maury and Pajuste (2005) examine the mechanism how the second and third large owner affect firm value (Maury and Pajuste, 2005), Luo et al. (2013) analyze how contest for control between other large owners and the largest owner affect firm value (Luo et al., 2013). For Chinese high-tech listed companies, the largest shareholders usually have a higher proportion of equity than other shareholders. They have both the same interests and different interests. For example, in terms of improving company performance and stock value, their interests may be consistent, and their goals may be inconsistent in the pursuit of short-term or long-term interests. The focus of the conflict between the largest shareholder and other major shareholders is that only the largest shareholder can take actions that harm other shareholders. Therefore, other large shareholders have strong intention to monitor the largest shareholders and prevent their opportunistic behavior. Nonetheless, other large shareholders usually support the behavior of the largest shareholders to improve the company’s performance. Therefore, we hypothesize:

*Hypothesis 2.1: In China, the shareholding ratio of other large share holders is positively related to the current performance.*

In addition to monitoring the opportunistic behavior of the largest shareholders and top managers, other large shareholders can also participate in company strategic decisions, such as green R&D investment and green marketing investment decisions. Undoubtedly, the risks of green R&D investment and green marketing investment are different, in the Chinese context, the risks of green R&D investment are usually much greater than green marketing investment. As mentioned above, the largest shareholders, due to their high shareholding ratios, they may like to invest in green marketing (because green marketing investment is usually less risky and can improve the company’s short-term performance). However, other large shareholders may not prefer green marketing investment (because they may worry that the company’s managers will take the opportunity to obtain private benefits). Although green marketing investment may help company to gain the short term returns, this return is limited and not very attractive to other large shareholders (because their shareholding ratio is not very high). On the other hand, how other large shareholders influence green R&D investment depends on their preference for the company’s strategy. Indeed, some studies have analyzed the impact of other large shareholders on R&D investment, for example, Tribo et al. (2007) have investigated the effect of the type and number of block-holders on R&D investment in Spanish (Tribo et al., 2007), and Hoskisson et al. (2002) examine the impact of different investors on innovation (Hoskisson et al., 2002), but these studies focus on the types of other large shareholders. Compared with the largest shareholder, other large shareholders have less control over the company, the relationship between other large shareholders and top managers can be regarded as a principal-agent relationship. Under the monitoring of the largest shareholder, other large shareholders cannot obtain benefits through opportunistic behavior. The only way they can obtain benefits is the development and growth of listed companies. Although green R&D investment may has failure risk, successful green R&D investment will greatly improve the company’s performance and create benefits for other large shareholders. This is because the company’s successful R&D in green technology will receive the attention and strong support of the government, which will further increase the trust and loyalty of customers to benefit in the company’s long-term development and earnings. In addition, compared with the largest owner, other large shareholders bear relatively smaller risk in green R&D investment, they can disperse this risk by portfolios. Therefore, other large shareholders may prefer innovation strategy, they may hope to realize the rapid growth and good performance of the company through green R&D investment.

Based on the above discussion, we argue that other major shareholders may have a more positive attitude and preference for R&D investment, more negative attitude and preference for marketing investment. Obviously, the shareholding ratio of other large shareholders determines their ability to influence the company’s strategic decision-making psychology, the higher the shareholding of other large shareholders, the stronger their ability to influence the company’s investing psychology, so we hypothesize:

*Hypothesis 2.2: In China, the shareholding ratio of other large shareholders is negatively related to the green marketing investment psychology.*

*Hypothesis 2.3: In China, ceteris paribus, the shareholding ratio of the other large shareholders is positively related to the green R&D investment psychology.*

### Equity Counterbalance

The equity structure of companies is one of the core mechanisms of corporate governance, to some extent, its distribution and characteristic determine the allocation of the control power and resources of companies (Zattoni, 2011). We have analyzed the impact of the largest shareholder and other major shareholders on strategic investment in green affairs and performance respectively. This is just a simple analysis. For example, when we analyze the effects of the largest shareholder, we assume that other factors remain unchanged. In other words, we did not analyze the possible impact of the interaction between the largest shareholder and other large shareholders on strategic investment in green affairs and company performance. As mentioned above, the interests of the largest shareholder and other major shareholders have both the same part and different parts. Therefore, they may have competition and cooperation/compromise in the strategic decision making process. The degree of competition and cooperation between the largest shareholder and other major shareholders depends on their relative power over the company, and their power over the company is mainly determined by the proportion of their shareholding. In the Chinese scenario, the largest shareholders generally have a high percentage of equity, have strong control over the company, and are prone to encroach on the interests of other shareholders and the company. Since the influence of small shareholders on the company is almost negligible, other large shareholders need to have the ability to monitor and restrict the largest shareholders’ behaviors, and to prevent the largest shareholder from doing anything that harms the interests of other shareholders or the company. Here, we put forward the concept of equity counterbalance, and define equity counterbalance as the ability of other large shareholders to monitor and restrict the largest shareholders.

In the context of unstable economic policies and market, there are many business investment opportunities and many investment traps. Because many business opportunities are time-bound, companies must make quick decisions and actions to seize them: quickly enter and exit after making a profit. As mentioned above, the largest shareholders’ shareholdings and risks are higher than others, and they prefer investments that can quickly improve the company’s short-term performance or generate “seeing benefits,” such as investing in short-term financial markets and marketing promotions, entering a traditional industry that can temporarily make a profit, etc. The higher the relative equity ratio of the largest shareholders, the stronger their control over the company, and the more likely the company is to act in accordance with their intentions. Conversely, if other large shareholders have higher relative equity, the stronger their ability to restrain the largest shareholder, the harder it is for the largest shareholder to quickly improve the company’s short-term performance.

Therefore, we believe that equity counterbalance has a negative impact on the company’s short-term performance. As we know, other large shareholders hold relatively small shares and have psychological states of defense and distrust to the largest shareholders. They may habitually oppose the proposals of the largest shareholders because they are always worried that these proposals may harm their interests. The obstacles of other large shareholders may reduce the efficiency of the largest shareholders in pursuing short-term benefits, for example, losing some short-term investment opportunities, some marketing investment proposals cannot be approved. Therefore, monitoring and restraint may reduce the company’s decision-making speed and lose certain business and market opportunities, which will negatively affect the company’s current performance. If we use the ratio of other large shareholders’ equity to the largest shareholder’s equity as a measure of equity counterbalance, we can propose the following hypothesis:

*Hypothesis 3.1: In China, equity counterbalance is negatively related to the current performance.*

The ability of other major shareholders to monitor and restrain the largest shareholder will not only directly affect the company’s short-term performance, but also affect the company’s strategic investment psychology, such as the R&D and marketing investment psychology.

As a resource-allocation way, high-tech companies’ R&D investment in green technologies has the characteristics of high risk, high investment and high earnings, the largest shareholders generally may not support green R&D investment, because green R&D investment generally does not immediately improve the company’s short-term performance, and there is also the risk of investment failure. At this time, their losses are greater than other shareholders. When largest shareholders cannot tolerate the risk of investment failure, they will strongly oppose the approval of this R&D. Unlike this, other large shareholders suffer less losses from green R&D investment failures because of their small shareholdings. Therefore, they may like to invest in high-risk and high-return green R&D projects. In practice, what strategic investment psychology a listed company have depends on the relative power between the largest shareholder and other major shareholders, the existence of other large shareholders will weaken the control/influence ability of the largest shareholder, dilute responsibilities among the large shareholders (Tribo et al., 2007), and reduce the discretion and self-serving behavior of executives (Tosi et al., 1997; Finkelstein and Boyd, 1998). The strategic decision of a Chinese-listed company is generally the result of struggle, negotiation or mutual compromise between various interest groups, especially between the largest shareholder and other major shareholders. The higher the ratio of other major shareholders’ equity to the largest shareholder’s equity, it indicates that the other large shareholders have greater ability to restrain and influence the largest shareholder. Therefore, the higher the degree of equity balance, the more the company’s strategic decision-making psychology can reflect the investment psychology of other major shareholders, taking into account the R&D investment preferences of other major shareholders, we believe that the equity counterbalances will have a positive impact on the R&D investment psychology. On the other hand, the largest shareholders are more willing to quickly improve the company’s short-term performance by increasing green marketing investment. However, because other large shareholders often distrust the largest shareholders and their agency executives, they often oppose excessive marketing budgets and limit some unreasonable marketing expenses, and because they worry that excessive marketing budgets may lead to corruption or abuse of marketing fees. Under the limited resources, green marketing investment may reduce the company’s green R&D investment capacity, and decrease the company’s ability to develop new green technologies and products and other major shareholders do not want this situation, and may try to reduce the company’s marketing investment sentiment. The higher equity counterbalance, the stronger other large shareholders’ influence ability to the company’s marketing investment psychology. Therefore, we believe that equity counterbalance has a negative effect on the company’s green marketing investment psychology.

Based on the above discussion, we hypothesize:

*Hypothesis 3.2: In China, equity counter balance is positively related to the green R&D investment psychology.*

*Hypothesis 3.3: In China, equity counterbalance is negatively related to the green marketing investment psychology.*

## Methodology

### Sample and Data

We choose China’s international high-tech companies to test our hypothesis. The companies include those in the areas of computer and communication technologies, instrument and meter manufacturing, electronic equipment manufacturing, bioengineering and pharmaceutical manufacturing. We collect the panel data from listed companies during the 2012–2017 period, in this period, there is a relatively stable industrial policy environment in China, we select sample companies by eliminating companies which are labeled by ST and ST^∗^(because ST or ST^∗^ firms’ operating and financial performance occur change abnormally, and their stocks may be ceased to trade in China stock exchanges). Finally, we got the balanced panel data of 342 listed high-tech companies. Our sample data mainly comes from CCER database in China, to verify the accuracy of the data, we repeatedly collect the same data from CSMA database, the result shows that our data has no obvious mistakes.

### Variables and Measures

#### Equity Structure

• The largest shareholders (EQ1).

We define the largest shareholder as the individual or organization holding the largest proportion of shares, and utilize the share holding ratio as measurement value.

• Other large shareholders (EQ2).

We define the second to tenth large shareholders as other large shareholders, and calculate the measuring value by the formula:

EQ2 = the sum of the share holding ratios of the second to tenth large shareholders.

• Equity counterbalance.

We define equity counterbalance as the ability of other large shareholders to restrict the largest shareholder, and calculate the measuring value by the formula:

EQB = EQ2/EQ1.

#### Strategic Investment Psychology

• Green R&D investment psychology (RD).

Chen V.Z. et al. (2014) have proposed a principal component approach to measuring investor sentiment (Chen H. et al., 2014), and Naseem et al. (2021) use similar methods to measure and test the influence of investor psychology on stock market behavior. However, due to the inability to collect multi-indicator data, we use the ratio of R&D expenditures in green technologies to the total sales income as indicator of green R&D investment psychology.

• Green marketing investment psychology (SE).

Similarly, considering the availability of second-hand data, We use the ratio of expenditures in green marketing activities to the total sales income as indicator of green marketing investment psychology.

#### Company Performance (ROA)

• We measure company performance through the Return on Assets, which is defined as the ratio of net profit before tax to total assets.

#### Control Variables

We control the variables which may influence strategic investment and performance, mainly including the following variables:

• Firm size (Size).

We measure firm size as the natural logarithm of the total assets.

• Asset-liability ratio (LEV).

Asset-liability ratio is calculated by total debts/total assets.

• Cash flow (CASH).

Cash flow is calculated by the cash flow generated in the business activities/total assets.

• Organizational slack (Slack).

Organizational slack is calculated by the sum of sales expenses, financial expenses and management expenses/sales income.

### Regression Model

To test the effect of the largest and other large shareholders on strategic investment and performance, we construct the following models (1), (2), and (3), we utilize model (1) and (2) to test the their impacts on green R&D and green marketing investment, model (3) to test their direct effects on performance and the mediating role of R&D and marketing investment.

(1) $${RD}_{i,t}=\alpha_{0}+\alpha_{1}{EQ1}_{i,t}+\alpha_{2}{EQ2}_{i,t}+\alpha_{k}\Sigma \text{Controls}+\mu_{i,t}$$

(2) $${SE}_{i,t}=\alpha_{0}+\alpha_{1}{EQ1}_{i,t}+\alpha_{2}{EQ2}_{i,t}+\alpha_{k}\Sigma \text{Controls}+\mu_{i,t}$$

(3) $${ROA}_{i,t}=\delta_{0}+\delta_{1}{\text{EQ1}}_{\text{i,t}}+\delta_{2}{\text{EQ2}}_{\text{i,t}}+\delta_{3}{\text{RD}}_{\text{i,t}}+\delta_{4}{\text{SE}}_{\text{i,t}}+\delta_{5}{\text{CASH}}_{\text{i,t}}+\delta_{6}{\text{LEV}}_{\text{i,t}}+\delta_{7}{\text{Size}}_{\text{i,t}}+\varphi_{\text{i,t}}$$

To test the effect of the equity counterbalance on strategic investment and performance, we construct the following models (4), (5), and (6), we utilize model (4) and (5) to test the impacts of the equity counterbalance on green R&D and marketing investment, model (6) to test their direct effects of the equity counterbalance on performance and the mediating role of green R&D and marketing investment.

(4) $$RD_{i,t}=\alpha_{0}+\alpha_{1}EQB_{i,t}+\alpha_{k}\Sigma \text{Controls}+\mu_{i,t}$$

(5) $$SE_{i,t}=\alpha_{0}+\alpha_{1}EQB_{i,t}+\alpha_{k}\Sigma \text{Controls}+\mu_{i,t}$$

(6) $${ROA}_{i,t}=\delta_{0}+\delta_{1}{EQB}_{i,t}+\delta_{2}{RD}_{i,t}+\delta_{3}{SE}_{i,t}+\delta_{4}{CASH}_{i,t}+\delta_{5}{LEV}_{i,t}+\delta_{6}{Size}_{i,t}+\varphi_{i,t}$$

where subscript *i*, *t* represents the measured value of variables of company *i* in year *t*, Σcontrols represents all of the control variables, in the equations, α_0_ and δ_0_ are intercepts, α_*i*_ and δ_*i*_ are the parameters to be estimate, μ_*i,t*_ and φ_*i,t*_ indicate the mixed random interference of the individual and time effects.

## Results

This section may be divided by subheadings. It should provide a concise and precise description of the experimental results, their interpretation as well as the experimental conclusions that can be drawn.

### Descriptive Statistics

To avoid the adverse influence of the extreme value, before the descriptive statistical analysis, we perform Winsorizing by the level of 1 and 99% for the variables with extreme observation values. By using the statistical software Stata 14, we report the results of the descriptive statistical analysis in Table 1.

**TABLE 1:** Means, standard deviations, min and max value.

Variable	Obs	Mean	Standard deviation	Min	Max
ROA	2,052	0.049	0.054	−0.145	0.210
RD	2,052	0.060	0.051	0.004	0.331
SE1	2,052	0.122	0.130	0.002	1.000
EQ1	2,052	0.318	0.138	0.004	0.692
EQ2	2,052	0.231	0.122	0.003	0.529
EQB	2,052	0.934	0.766	0.025	6.562
CASH	2,052	0.045	0.060	−0.147	0.206
LEV	2,052	0.337	0.189	0.038	0.845
Slack	2,052	0.261	0.156	0.044	0.775
Size	2,052	21.822	1.018	19.729	24.666

The data shows that the average equity ratio of the largest and other large shareholders are 0.318 and 0.231, this means that, on average, the largest shareholder holds more shares than other large shareholders. The minimum value of EQ1 is 0.4% and the maximum value is 69.2%, and the standard deviation is 14%, these observation numbers manifest that the largest shareholder has a high shareholding ratio, there still exists dominant shareholders in the China’s high-tech listed companies. The average value of EQB is 0.934, these numbers show that the shareholding ratio sum of other large shareholders are close to the one of the largest shareholder on average.

### Correlations Analysis

Before the regression analysis, in order to have a preliminary understanding of the relationship between the research variables, we conducted a correlation analysis on all variables with Stata 14.0 software, and the results are shown in Table 2.

**TABLE 2:** Correlations.

	ROA	RD	SE	EQ1	EQ2	EQB	CASH	LEV	Slack
ROA	1.00								
RD	−0.13***	1.00							
SE	0.26***	−0.01	1.00						
EQ1	0.19***	−0.09***	0.07***	1.00					
EQ2	0.08***	0.08***	0.02	−0.26***	1.00				
EQB	−0.05*	0.14***	−0.02**	−0.62***	0.73***	1.00			
CASH	0.48***	−0.07***	0.15***	0.11***	0.01	−0.04*	1.00		
LEV	−0.36***	−0.21***	−0.19***	−0.10***	−0.23***	−0.08***	−0.20***	1.00	
Slack	0.03	0.34***	0.81***	−0.02	0.02	0.04*	−0.01	−0.15***	1.00
Size	0.09***	−0.13***	−0.05*	0.00	−0.08***	−0.03	0.06***	0.43***	−0.16***

*P < 5%, **P < 1%, and ***P < 0.1%.

Table 2 shows that the EQ1 is significantly related to ROA, RD, and SE. EQ2 is significantly related to ROA and RD, EQB is significantly related to ROA, RD, and SE.

In this paper, the Pearson correlation coefficients between the most variables are almost below 0.5, this indicate that there is no obvious multicollinearity problem in our study, the sample data are suitable for multiple regression analysis.

### Regression Analysis

#### The Largest and Other Shareholders

To prevent the occurrence of “pseudo-regression,” it is necessary to ensure the stability of the data. Therefore, we examine the stability of the data by using the HT test method, for the sake of short-panel data, we conduct unit root tests for all of the study variables by Stata Command (xtunitroot ht), the results indicate that all variables have no unit root, this mean that our study variables data are stable. We use Model (1) and (2) to test hypothesis 1.2 and 1.4, and hypothesis 2.2 and 2.3. Model (3) is used to test hypothesis 1.1 and 2.1, hypothesis 1.2 and 1.5. The results are shown in Table 3.

**TABLE 3:** Stepwise regression result.

Model	(1)	(2)	(3)	(3)	(3)
Variables	RD,RE	SE,RE	ROA,FE	ROA,FE	ROA, FE
EQ1	−0.026*** (−3.37)	0.038*** (3.25)	0.052*** (5.78)		0.042*** (4.89)
EQ2	0.017^+^ (1.84)	0.015 (1.03)	0.024* (2.46)		0.024* (2.48)
RD				−0.23*** (−9.84)	−0.219*** (−9.39)
SE				0.042*** (4.04)	0.04*** (3.92)
CASH	−0.019 (−1.52)	0.063*** (3.38)	0.221*** (13.35)	0.21*** (12.86)	0.208*** (12.79)
LEV	−0.034*** (−5.74)	−0.059*** (−6.57)	−0.102*** (−13.99)	−0.115*** (−16.45)	−0.109*** (−15.02)
Slack	0.153*** (20.42)	0.507* (44.52)			
Size	0.000 (−0.10)	0.008*** (4.70)	0.011*** (8.61)	0.010*** (8.26)	0.011*** (8.48)
cons	0.040 (1.57)	−0.185*** (−4.77)	−0.194*** (−6.91)	−0.14*** (−5.25)	−0.169*** (−6.18)
N	2052	2052	2052	2052	2052
R2	0.206	0.697	0.340	0.37	0.380
chi2	480.39***	2036.86***	546.21***	648.60***	684.26***

The number in the brackets is z value, ^+^P < 10%, *P < 5%, **P < 1%, and ***P < 0.1% (two-tailed tests for all variables).

First, we use panel-data regression to test the influence of the largest and other large shareholders on R&D and marketing investment. In the equation (1), we control all variables which may influence R&D investment, such as firm size, leverage ratio, cash flow, and organizational slack. The results show that the shareholding ratio of the largest shareholder has negative effect on R&D investment (α1 = −0.026, *p* < 0.001), and the shareholding ratio of other large shareholders has positive effect on R&D investment (α1 = 0.017, *p* < 0.1). Therefore, hypothesis 1.4 is supported by sample data. If *P* < 0.1 is accepted, then hypothesis 2.3 is also supported by sample data. The regression results of Model (2) indicate that the shareholding ratio of the largest shareholder is positively related to marketing investment (α1 = 0.038, *p* < 0.001), but the regression coefficient of EQ2 is not significant, therefore, hypothesis 1.2 is supported by empirical data, and hypothesis 2.2 is not supported by empirical data. From the first-step regression results of Model (3) (see Table 3), we can find the regression coefficients of EQ1 and EQ2 are significant (δ1 = 0.052, *p* < 0.001; δ2 = 0.024, *p* < 0.05). This result shows that the largest shareholder and other large shareholders alone have a positive impact on the company’s performance without considering their interactions. Therefore, hypothesis 1.1 and hypothesis 2.1 are both supported by sample data.

Secondly, we further use Model (3) to test the mediating effects of green R&D and marketing investment on the relationship between the equity ratio of the largest shareholder and company performance. From the second-step regression results of Model (3) (see Fifth column in Table 3, we can find that R&D investment has significant and negative influence on company performance (δ3 = −0.23, *p* < 0.001), marketing investment has significant and positive influence on company performance (δ4 = 0.042, *p* < 0.001). From the third-step regression results of Model (3) (see Sixth column in Table 3, we can find that the regression coefficients of EQ1 and EQ2 are still significant (δ1 = 0.042, *p* < 0.001; δ2 = 0.024, *p* < 0.05), and the regression coefficients of R&D and marketing investment on company performance are also significant (δ3 = −0.219, *p* < 0.001; δ4 = 0.04, *p* < 0.05). Compare to the results of Fourth column of Table 3, the regression coefficient of EQ1 changes noticeably (δ1 alter from 0.052 to 0.042, and Z-value change from 5.78 to 4.89), the regression coefficient of EQ2 almost does not change. Referring to the method of testing the mediation effect by regression analysis, and comprehensively consider the results of the five regression equations in Table 3, we can determine that R&D investment has a negative intermediary role in the relationship between the largest shareholder’s shareholding ratio and the company’s performance, and marketing investment has a positive intermediary role in the relationship between the largest shareholder’s shareholding ratio and the company’s performance. Therefore, hypothesis assumptions 1.3 and 1.5 are supported by empirical data.

Similarly, we use stepwise regression to test the effects of equity counterbalance on R&D investment, marketing investment and company performance. The results are shown in Table 4.

**TABLE 4:** Stepwise regression result.

Model	(4)	(5)	(6)	(6)	(6)
Variables	RD,RE	SE,RE	ROA,RE	ROA, RE	ROA, RE
EQB	0.005** (3.56)	−0.003 (−1.43)	−0.003^+^ (−1.82)		−0.001 (−0.83)
RD				−0.23*** (−9.84)	−0.229*** (−9.71)
SE				0.042*** (4.04)	0.041*** (4.03)
CASH	−0.020 (−1.67)	0.063*** (3.39)	0.225*** (13.44)	0.21*** (12.86)	0.210*** (12.84)
LEV	−0.031*** (−5.26)	−0.063*** (−7.00)	−0.11*** (−15.49)	−0.115*** (−16.45)	−0.116*** (−16.44)
Slack	0.153*** (20.46)	0.505* (44.36)			
Size	0.000 (−0.29)	0.007*** (4.19)	0.011*** (8.46)	0.010*** (8.26)	0.011*** (8.31)
cons	0.034^+^ (1.43)	−0.144*** (−3.96)	−0.164*** (−5.90)	−0.14*** (−5.25)	−0.143*** (−5.28)
N	2052	2052	2052	2052	2052
R2	0.206	0.694	0.337	0.37	0.371
chi2	478.48***	2019.01***	506.06***	648.60***	649.77***

The number in the brackets is z value, ^+^P < 10%, *P < 5%, **P < 1%, and ***P < 0.1% (two-tailed tests for all variables).

First, we use Model (4) and (5) to test the effects of equity counterbalance on R&D and marketing investment. The results show that equity counterbalance has a positive impact on R&D investment (α1 = 0.005, *p* < 0.01), and has no significant effect on marketing investment (α1 = −0.003, *p* < 0.2). Therefore, hypothesis 3.2 is supported by empirical data, and hypothesis 3.3 is not supported by empirical data. We use Model (6) to test hypothesis 3.1, hypothesis 3.4, and hypothesis 3.5. From the Table 4, we can find that the regression coefficient of equity counterbalance to company performance is −0.003, and *p* < 0.1, if we accept such statistical significance, the hypothesis that equity counterbalance has a negative impact on the current company performance is supported. In other words, hypothesis 3.1 is supported by sample data.

When we continue to add RD and SE to test hypothesis 3.4 and hypothesis 3.5, from the results in the sixth column of Table 4, we can find that the regression coefficient and significance of EQB on ROA are reduced (δ1 = −0.001, *p* = 0.407), because the effect of equity counterbalance on marketing investment is not significant, the mediating effect of marketing investment may not exist, and hypothesis 3.5 is not supported. On the other hand, we believe that equity counterbalance mainly affects company performance through R&D investment. In other words, equity counterbalance has a certain negative impact on the company’s current performance through its positive impact on R&D investment. Therefore, hypothesis 3.4 is supported by empirical data.

## Discussion and Conclusion

### Conclusion

Integrating agency and stewardship theory, this study examines the relationships among the equity structure, strategic investment psychology in green affairs and company performance. Our research results show that:

(1) The largest shareholder’s shareholding ratio has a positive effect on marketing investment psychology and a negative impact on R&D investment psychology;
(2) Other large shareholders’ shareholding ratio are positive related to R&D investment psychology;
(3) R&D investment psychology has a negative effect and marketing investment psychology has a positive influence on the current performance;
(4) Equity counterbalance is positive related to R&D investment psychology and has a negative effect on the current performance.

Our findings indicate that the largest shareholder has a psychological tendency to prefer marketing investment and seek short-term performance, and other large shareholders have a psychological tendency to prefer R&D investment and pursue long-term performance. When their shareholding ratio is higher, their influence on the companies’ strategic investment psychology is greater. The findings also show that the marketing-oriented psychology helps to improve the company’s short-term performance, and the R&D-oriented psychology reduces the company’s current performance.

Finally, our findings show that the restrictive role of other large shareholders on the largest shareholders may be mainly reflected in high-risk strategic decisions, such as green R&D investment.

### Theoretical Implications

First, our study contributes to the literatures that study the relationship between equity structure and R&D investment. Previous empirical studies have found that ownership concentration has a positive impact on R&D investment (Baysinger et al., 1991; Lee and O’neill, 2003). Similarly, Tribo et al. (2007) report that the number of control shareholders is negatively related to the firm’s R&D investment intensity, which supports the above finding from another perspective. Different from the above findings, our empirical results show that if ownership is more concentrated in the largest shareholder, it will have a negative impact on green R&D investment. On the contrary, increasing the shareholding ratio of other large shareholders will have a positive impact on green R&D investment. Our findings further show that the relationship between equity structure and R&D investment will change with different cultural and institutional environments. Thus, our study contributes to the literature by providing a new empirical evidence. On the other hand, we add marketing investment to the above research framework, which has further expanded the scope of this research stream.

Second, our study also contributes to the literatures that analyze the relationship between equity structure and firm performance. Many studies have examined the direct influence of equity structure on performance (Han and Suk, 1998; Kapopoulos and Lazaretou, 2007; Fattoum-Guedri et al., 2018). However, few literatures pay attention to the internal mechanism by which equity structure affects performance, and our study has enriched the research in this field. Zhang et al. (2014) reported that ownership concentration has positive influence on performance through the mediating role of R&D investment. Our research finds that green R&D investment has negative and positive mediating effect in different equity structure-performance relationship. In addition, our research suggests that strategic investment (such as green R&D and green marketing investment) may be an important intermediary variable in the equity structure-performance relationship, this opens a new window for the study of the “internal mechanism of ownership structure affecting company performance.”

### Managerial Implications

Our study has some managerial implications for the practices of equity structure governance policies. Our research results show that: in the Chinese context, increasing the shareholding of the largest shareholder is good for the company’s short-term performance, but it is not good for the company’s innovation and sustainable development. Increasing the relative shareholding ratio of other large shareholders to the largest shareholder is not good for the company’s short-term performance, but it is beneficial to the company’s innovation and sustainable development. In the context of global advocacy of green and sustainable development, manufacturing companies need to continuously carry out technological innovations to improve energy-saving and emission-reduction production capabilities. This requires companies to continuously increase investment in the design, production and marketing of environmentally friendly products. The findings of this study has implications on how manufacturing companies can adjust their shareholding structure to promote green investment in correct aspect and dimension.

If high-tech listed companies are in a state of relatively stable income but insufficient growth, increasing the shareholding ratio of other major shareholders or reducing the shareholding ratio of the largest shareholder will help the company increase its green R&D investment, benefit the company’s technology and product innovation, and promote the company long-term development.

### Limitations and Future Research

Our research conclusions are mainly based on high-tech listed companies, we do not know that whether these conclusions are applicable to non-listed companies or companies in other industry. Future studies can further examine the results by using samples of non-listed companies or companies in other industry. In addition, due to the inability to collect relevant data, our measurement of R&D investment psychology and marketing investment psychology is relatively simple, and future research can use a multi-index sentiment index measurement method.

## Data Availability Statement

The datasets generated for this study are available on request to the corresponding author.

## Author Contributions

LG led the project, acquired the funding support, and was the major author for the first draft. K-LL was responsible for constructing the overall research framework and reviewed and thoroughly edited the original manuscript. C-FL was responsible for empirical data analysis. L-TZ had completed the modification based on the reviewers’ revision proposal.

## Conflict of Interest

The authors declare that the research was conducted in the absence of any commercial or financial relationships that could be construed as a potential conflict of interest.

## Publisher’s Note

All claims expressed in this article are solely those of the authors and do not necessarily represent those of their affiliated organizations, or those of the publisher, the editors and the reviewers. Any product that may be evaluated in this article, or claim that may be made by its manufacturer, is not guaranteed or endorsed by the publisher.
